# Chimpanzee females queue but males compete for social status

**DOI:** 10.1038/srep35404

**Published:** 2016-10-14

**Authors:** Steffen Foerster, Mathias Franz, Carson M. Murray, Ian C. Gilby, Joseph T. Feldblum, Kara K. Walker, Anne E. Pusey

**Affiliations:** 1 Department of Evolutionary Anthropology, Duke University, USA; 2 Leibniz Institute for Zoo and Wildlife Research (IZW) in the Forschungsverbund Berlin e.V., Germany.; 3 Department of Anthropology, George Washington University, USA; 4 School of Human Evolution and Social Change, and Institute of Human Origins, Arizona State University, USA.

## Abstract

Dominance hierarchies are widespread in animal social groups and often have measureable effects on individual health and reproductive success. Dominance ranks are not static individual attributes, however, but instead are influenced by two independent processes: 1) changes in hierarchy membership and 2) successful challenges of higher-ranking individuals. Understanding which of these processes dominates the dynamics of rank trajectories can provide insights into fitness benefits of within-sex competition. This question has yet to be examined systematically in a wide range of taxa due to the scarcity of long-term data and a lack of appropriate methodologies for distinguishing between alternative causes of rank changes over time. Here, we expand on recent work and develop a new likelihood-based Elo rating method that facilitates the systematic assessment of rank dynamics in animal social groups, even when interaction data are sparse. We apply this method to characterize long-term rank trajectories in wild eastern chimpanzees (*Pan troglodytes schweinfurthii*) and find remarkable sex differences in rank dynamics, indicating that females queue for social status while males actively challenge each other to rise in rank. Further, our results suggest that natal females obtain a head start in the rank queue if they avoid dispersal, with potential fitness benefits.

Dominance hierarchies are widespread in animal social groups and an individual’s dominance rank can have broad consequences for its health and reproductive success^1^,^2^,^3^,^4^,^5^,^6^. However, individual rank positions (i.e., absolute position in the hierarchy) generally do not remain stable across the life span, but instead are influenced by two independent processes: 1) changes in hierarchy membership that shift existing rank positions upward or downward without changing relative rank relationships, and 2) changes in relative (i.e., dyadic) rank relationships that are triggered by successful challenges of higher-ranking individuals. Understanding which of these two processes dominates the long-term dynamics of rank trajectories can provide insights into fitness benefits of intra-sexual competition.

In many dominance hierarchies, relative rank relationships remain very stable across time, maintained by individual differences in age [e.g., male boat-tailed grackles (*Quiscalus major*)^7^, male rats (*Rattus norvegicus*)^8^, female elephants (*Loxodonta africana*)^9^, female mountain goats (*Oreamnos americanus*)^10^], body mass [e.g., male clown fish (*Amphiprion percula*)^11^], or through social support [e.g., female Japanese macaques (*Macaca fuscata*)^12^, female yellow baboons (*Papio cynocephalus*)^13^, male and female spotted hyenas (*Crocuta crocuta*)^14^,^15^]. In such systems, absolute rank positions change mainly by entry of new individuals into the hierarchy through immigration or maturation and exit of others by death or dispersal. By contrast, in other cases successful challenges of higher-ranking individuals occur, which lead to dynamic changes in relative rank relationships over time [e.g., female hover wasps (*Liostenogaster flavolineata*)^16^, male long-tailed macaques (*Macaca fascicularis*)^17^, male yellow baboons^18^]. Such challenges might be related to aging, disease or injury in the higher-ranking individual, or factors such as growth and social support that increase the fighting ability of the lower-ranking individuals.

Species and sex differences in dominance rank dynamics likely relate to the tradeoff between potential benefits and costs of aggressive competition^6^,^19^. However, the lack of systematic approaches to the study of rank trajectories across a wide range of study systems (i.e., social groups, populations, species) leaves open many questions about the factors that explain variation in the long-term stability of rank relationships and the processes that mediate individual rank trajectories across the life span. This gap in understanding is caused not only by the difficulty of obtaining rank assessments of identified individuals throughout their adult lives, but also by the challenges associated with accurately quantifying the rank relationships at any point in time. Traditional matrix-based methods for calculating dominance rank^20^,^21^ often require aggregating data over arbitrary time periods, which can make it difficult to keep track of changes in group composition across matrices and distinguish between the influence of demographic change and successful rank challenges on rank changes over time.

As an alternative, Elo rating^22^ can overcome several shortcomings of matrix-based approaches to quantifying rank positions^23^,^24^. This method calculates and continuously updates so called Elo scores at each observed interaction, therefore avoiding the need for selecting arbitrary time windows. Pairwise differences in these Elo scores are used to obtain winning probabilities for each individual against each other individual, and can be used to rank individuals in a dominance hierarchy. Previous implementations of the Elo rating method assign all individuals the same initial Elo score before their first interaction (e.g., refs 23, 24 and 25, but see the “Elo-Rating” package for the possibility of more complex predefined rules for the assignment of initial Elo scores^26^). In these approaches, any incorrect initial assignment is corrected over time based on observed interactions. However, these initial assignments might be problematic when used with sparse interaction data, because 1) apparent rank changes that emerge because wrongly assigned initial Elo scores are being corrected over time cannot easily be distinguished from real rank changes that arise from successful challenges of higher-ranking individuals, and 2) the initial correction process can take a long time. Consequently, rank trajectories and their biological basis may be misrepresented when using the standard Elo rating method, despite its advantages over matrix-based approaches.

Here we develop an extension of the Elo rating method that addresses the shortcoming described above. For that purpose, we extend a recently developed modification of the Elo rating method that uses maximum likelihood fitting of Elo model parameters^25^. We apply this new method to characterize long-term rank trajectories in wild eastern chimpanzees (*P. troglodytes schweinfurthii*), with emphasis on sex differences in the dynamics of rank relationships. Male chimpanzees are philopatric, more gregarious than females, and compete aggressively for positions in a (usually linear) hierarchy^27^,^28^. Young males tend to rise in rank as they successfully challenge higher-ranking individuals, achieve their top rank as prime adults (roughly at 21 years of age at Gombe), and decline in rank after being successfully challenged by younger males^28^,^29^. In contrast to males, female chimpanzees tend to emigrate to another community after reaching sexual maturity, although the degree of female dispersal varies between study sites^30^. The generally low frequency of decided agonistic interactions among females has made their rank relationships difficult to characterize^28^,^31^,^32^,^33^,^34^, although findings from previous studies indicate that rank tends to increase with female age^34^,^35^,^36^,^37^,^38^. Whether and how successful rank challenges influence female chimpanzees’ long-term rank trajectories, however, is currently not understood.

Using our extended Elo rating method to obtain optimal rank estimates for both male and female chimpanzees, we conduct the first robust test of the hypothesis that, while male rank trajectories are characterized by successful challenges, female chimpanzee rank trajectories are mainly determined by changes in hierarchy membership where individuals queue for social status^36^. In particular, we test the related predictions that (1) females tend to enter the hierarchy at low dominance ranks, (2) successful rank challenges among females are rare and/or temporary, and (3) females mainly rise in rank when senior high-ranking females die.

## Methods

### Data collection

Data for this study come from the Kasekela community of chimpanzees at Gombe National Park, Tanzania^28^. Between 1969 and 2000, social interactions were recorded between chimpanzees at an artificial feeding area where they were sometimes provisioned with bananas^27^. In addition, since 1970, field assistants (as well as graduate students over more recent periods) have conducted almost daily all-day focal observations on identified individuals, during which they also take detailed *ad libitum* notes on social interactions among other members of the focal individual’s party. These notes include pant-grunt vocalizations, which are formal indicators of subordinate status among chimpanzees^33^,^39^.

For modeling rank trajectories, we used all dyadic pant-grunt interactions extracted from the focal and *ad libitum* notes for the period 1969–2013 (females) and 1978–2011 (males), and for which a dominant and subordinate individual could be clearly identified. To maximize the number of decided agonistic interactions among adult females, we also included aggressive interactions for which a clear winner could be determined, when possible (availability of aggression data from *ad libitum* notes is incomplete and not evenly spread across years). As pant-grunt and aggressive interactions could have different directional consistencies, we repeated our analyses without aggression data. Although both model fit and predictive accuracy of estimated ranks worsened when decided aggressions were excluded, we obtained the same qualitative results.

To facilitate model fitting and interpretation, we excluded individuals who did not have at least one win and one loss over the length of the study period. We applied this requirement iteratively until all individuals remaining in the dataset met the inclusion criterion (excluding 13 females and 4 males). The final dataset consisted of 1,015 decided agonistic interactions from 44 adult females (46.1 ± SD 53.6 interactions per female, range: 2–272), and 2,741 interactions from 22 adult males (249.2 ± SD 244.5 interactions per male, range: 23–1,034). We considered females adult if they were 12 years or older at the time of the interaction, as the earliest age at first birth for known-aged females at Gombe is between 11 and 12 years^40^. The age of immigrant females, if unknown, was estimated based on body size, presence of full sexual swellings, and the average age of dispersing females of known age from the study community. For males, we used an age cutoff of 15 years. This cutoff is more conservative than the one used in some previous studies at Gombe that have used 12 years^41^,^42^, based on reproductive maturity. However, full size and social maturity is generally not achieved until males are 15 or 16 years old, at which time they become fully integrated into the male hierarchy and successfully challenge other adult males^28^.

Strong social preferences between mothers and their daughters have recently been documented empirically^43^, and may be associated with maternal support in initial rank acquisition for non-dispersing daughters of resident females. Therefore, as a potential predictor of rank at hierarchy entry, we classified natal females based on whether their mother was alive and present in the same community when they reached 12 years of age. We expect natal females whose mothers were alive at hierarchy entry to obtain higher initial rank positions compared to immigrant females and orphaned natal females. Mother-daughter relationships were determined from long-term genealogical records.

### Rank assessments

#### The original Elo method

The Elo rating method was originally developed for calculating cardinal dominance ranks, which are measured by so-called ‘Elo scores’. For all individuals the initial Elo score is set to a predefined constant *Elo_init*, e.g. 1000. Individual Elo scores are then updated at each observed interaction. Specifically, winners receive a “winner’s bonus”, which increases their Elo score, and the losers pay a “loser’s tax”, which decreases their Elo score. In a given dyadic interaction, the winner’s bonus and the loser’s tax are equal. Their absolute value depends on two quantities: (i) the predicted probability (prior to the encounter) that the winner wins and (ii) a predefined constant *k* (see details below).

The calculation of winning probabilities is based on an s-shaped function that results in a higher probability of winning for the individual with the higher Elo score. For instance Franz *et al*.^25^ assumed that given the Elo scores *Elo*_*A*_ and *Elo*_*B*_ of two individuals *A* and *B,* the probability *p*_*A,B*_ that *A* wins against *B* is given by a sigmoid function:


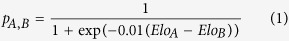


The execution of the Elo rating method is based on a consecutive evaluation of all observed agonistic interactions. For each interaction new Elo scores are calculated for the individuals involved in the interaction. For all individuals *C* who do not participate in an interaction *i* the Elo score of their previous interaction is maintained (*Elo*_*C,i*_ = *Elo*_*C*,*i-1*_). In contrast, for the two interacting individuals, A and B, new Elo scores are calculated. Specifically, in an interaction *i* with a predicted winning probability *p*_*A,B,i*_ with which individual *A* wins against *B*, new Elo scores *Elo*_*A*,*i*_ and *Elo*_*B*,*i*_ are calculated by:


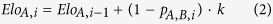



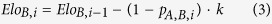


These equations imply that if the observed outcome was highly expected (i.e. when *p*_*A,B,i*_ is close to 1), Elo scores do not change much. In contrast, when the outcome was very unexpected (i.e. when *p*_*A,B,i*_ is close to 0) Elo scores change maximally. The maximum amount of change is determined by the value of the constant *k*, an input parameter set by the user.

#### Extensions of the original Elo rating method

We implemented the following extensions of the Elo rating method in our analyses:

##### Maximum likelihood fitting

According to a recently described maximum likelihood approach^25^, we calculated the overall log-likelihood of the observed data given a value of *k* (or a set of additional model parameters) based on winning probabilities (as calculated in equation 1) for all interactions *i*. We implemented maximum likelihood fitting with the function ‘optim’ in R^44^. As indicated by equations 2 and 3 the estimated value of *k* symmetrically applies to all interactions in the data set (excluding a burn-in period, see section ‘Model fitting’).

##### Forcing entry at the bottom

To assess whether individuals always enter the hierarchy at the bottom, we fitted a model that constrains initial Elo scores *Elo_init* for each new individual to the minimum Elo score of all adult individuals that were present in the community at the time of entry into the hierarchy (i.e., immigrating into the community or reaching 12 (females) or 15 (males) years of age for natal individuals).

##### Fitting initial Elo scores

Finally, instead of constraining all initial Elo scores *Elo_init* to specific values we allowed these scores to vary among individuals. For that purpose, we fitted the initial scores of all individuals using the described maximum likelihood approach. In this approach, all individual starting values were unconstrained and estimated jointly, together with estimating the value of *k* that most consistently predicts wins and losses in our dataset.

### Analyses

Our analyses consisted of two main parts. First we fitted alternative Elo models (separately for each sex) to our data to obtain a set of individual rank trajectories that best describe the observed data. Second, we examined these rank trajectories to assess the relative importance of successful rank challenges and changes in hierarchy composition on female rank trajectories, and tested our predictions about the determinants of female rank positions at time of hierarchy entry.

The datasets and R script supporting this article are available from the Dryad Digital Repository: http://dx.doi.org/10.5061/dryad.r4g74.

#### Model fitting

For both sexes we separately fitted the above-described models, all of which included fitting parameter *k* (equations 2 and 3), but differed in the way in which individuals entered the hierarchy. In the first model we followed the conventional practice of assigning all individuals the same initial Elo score. In the second model we forced individuals to enter the hierarchy at the bottom. Finally, in the third model we fitted the initial Elo scores of all individuals, i.e. we used maximum likelihood to determine the optimal starting Elo score for each individual.

To facilitate the implementation of model fitting we substituted *k* in equations 2 and 3 with exp(*β*), which ensures that during the model fitting for any estimated value of *β* the value of *k* is positive and therefore the Elo score of a winner never decreases and the Elo score of a loser never increases. For Models 1 and 2 we set a burn-in period to account for the fact that in these models all initially present individuals have the same Elo score, and as described above the Elo rating methods requires time to infer rank order based on observed interactions. For both models we set the burn-in period to the first 100 recorded interactions, and excluded them from the likelihood calculation; we set *k* to 100 for all interactions during the burn-in period. Model 3 does not require any burn-in period because initial Elo scores are fitted for individuals who were present initially and for those who entered later in time. To base model comparisons on the same data points, we therefore excluded the first 100 interactions when fitting Model 3. We compared model fit with the Akaike Information Criterion (AIC)^45^. In addition, we calculated for each model the proportion of all interactions in which a higher ranking individual won against a lower ranking individual, which indicates how well the model-derived Elo scores correctly predict observed interactions.

#### Investigation of female rank trajectories

To investigate female rank trajectories, we fitted the best model to the entire dataset, including the first 100 interactions that were excluded for the purpose of model comparisons (see above). Refitting of the model resulted in small changes in estimated parameters but did not alter the estimated rank dynamics, while increasing our sample size for the analysis of rank trajectories and their determinants.

To quantify the relative importance of successful rank challenges for the dominance rank trajectories of female chimpanzees we calculated the number of switches in ordinal ranks (which were calculated based on Elo scores) that occurred after individuals established their initial ranks. To assess the impact of changes in group composition on hierarchy dynamics we first investigated where individuals entered and exited the hierarchy. If female chimpanzees indeed queue for dominance ranks then individuals who enter should generally enter at or near the bottom, and individuals who leave the hierarchy should generally have a higher rank.

To quantify ranks at entry and exit we used two different measures. The first measure is the relative ordinal rank that is derived from the order of Elo scores that are normalized to a minimum of zero (representing the lowest ranking individual) and maximum of one (representing the highest ranking individual). This is an easily interpretable measure that simply quantifies the proportion of resident individuals that the entering individual ‘jumped over’. However, this measure ignores the possibility that dominance relationships are not necessarily completely unidirectional. For instance, an individual who enters at the bottom of the hierarchy might be strongly dominated by the lowest ranking resident, which would result in large Elo score differences and very low winning chances of the entering individual. Conversely, weak domination of a newly entered individual would be reflected in small Elo score differences and thereby winning chances against the lowest ranking resident that are closer to 0.5 (50%). Relative ordinal ranks ignore this kind of variation, because all individuals who enter at the bottom of the hierarchy have an initial relative ordinal rank of zero. Using relative Elo scores (scaled between 0 and 1) instead of relative ordinal ranks also does not solve this problem, because all individuals who enter at the bottom would have the same relative Elo score of zero.

To appropriately capture variation in winning probabilities beyond the level of ordinal ranks or relative Elo scores, we developed a new cardinal ranking metric based on a generalization of proportional ordinal ranks. In an ordinal ranking system, all dyadic relationships are binarized so that one individual is dominant and the other one is subordinate, which can be used to calculate, for each individual, what proportion of individuals in the group it dominates. The Elo rating method allows us to replace this binary approach with one that captures variation in the strength of rank differences. In the Elo rating method, winning probabilities (equation 1) quantify the degree to which one individual dominates the other. Instead of summing up the number of dominated individuals, we can sum up all pairwise winning probabilities *p*_*i,j*_ (see equation 1) for an individual *i* to obtain a quantity *D*_*i*_ on any given day *d*:


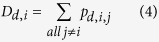


This measure can be interpreted as the expected number of dominated individuals. For example, in a hierarchy of three individuals, if the *γ* individual wins against the *β* individual with a 0.4 chance and against the *α* individual with a 0.1 chance, then the expected number of individuals that the *γ* individual can dominate is 0.5. We then calculated relative cardinal ranks for each individual *i* by dividing *D*_*i*_ by the count of all other individuals *j* (excluding individual *i*) present in the group at the time of entry or exit of *i*. If all dominance relationships are predicted to be perfectly directional (i.e. all estimated winning chances based on Elo score differences are 0 or 1) then the relative cardinal ranks will be identical to relative ordinal ranks.

To investigate the causes of variation in entry ranks among females, we used the results of Model 3 to quantify how often individuals did not enter at the lowest rank, i.e. how often they jumped the queue. In addition, we quantified how far individuals jumped. We compared how these quantities differed among three classes of individuals: 1) immigrants who entered after the beginning of the observation period (excluding one individual whose mother was a temporary visitor to the Kasekela community), 2) natal females whose mother died before the female entered the adult hierarchy, and 3) natal females whose mother was still present at hierarchy entry.

To quantify queue jumps at hierarchy entry we again used ordinal and cardinal ranks as described above, but without normalizing them. Thus, the first measure we used is the number of lower ranking individuals in the group at the date of entry (i.e. either date of immigration or date of reaching age of 12 years for natal females). The second measure we used is the expected number of dominated individuals at entry (equation 4).

## Results

### Model comparisons

**Figure 1:**
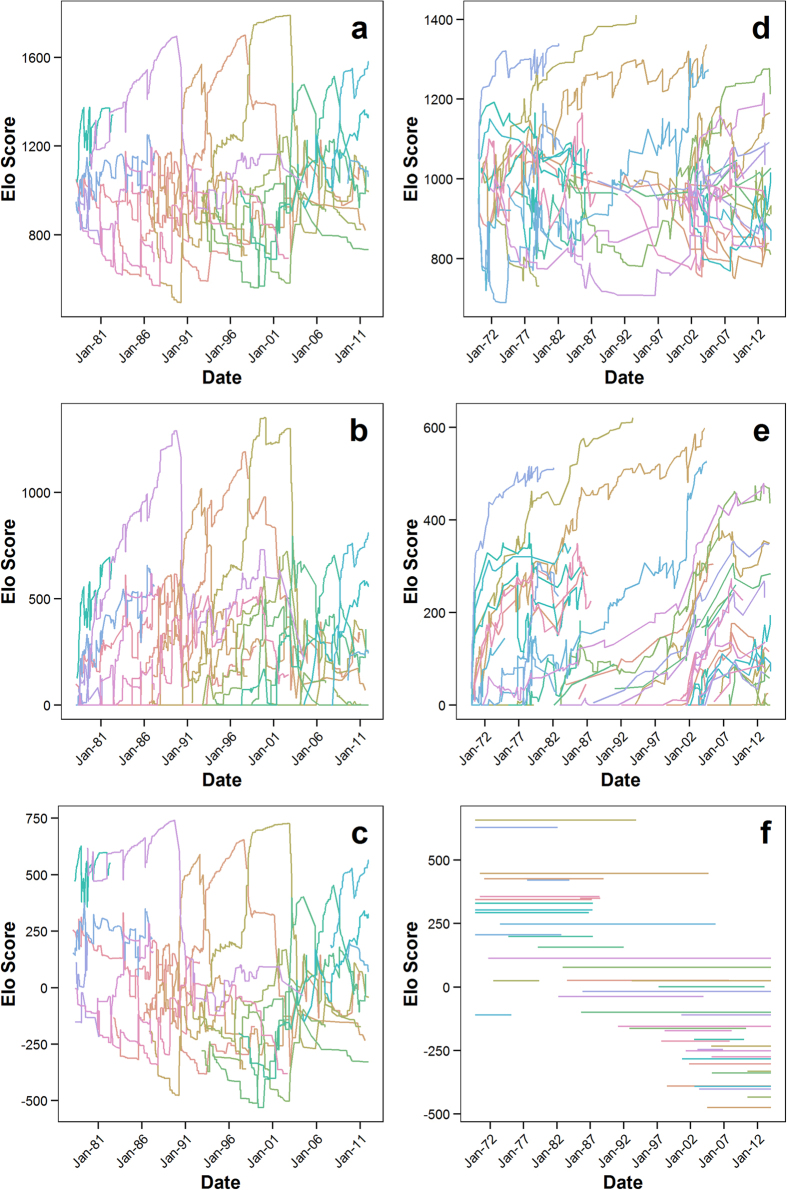
Illustration of estimated rank trajectories in males (**a–c**) and females (**d–f**) corresponding to models 1–3 in Table 1.

For males, the preferred model was Model 1, in which all males enter the hierarchy with the same Elo score (Table 1). As expected, in all fitted models the male hierarchy was characterized by frequent relative rank changes due to successful challenges (Fig. 1a–c). For females, the most preferred model was Model 3 (Fig. 1f), which included fitting of initial Elo scores (Table 1). In this model the estimated value of *k* was effectively zero. Thus, outcomes of observed agonistic interactions are not used to further update Elo scores, and individual Elo scores remain constant at their initial, estimated Elo scores. This finding indicates that the frequent rank changes observed in Model 1 (Fig. 1d) and Model 2 (Fig. 1e) are largely an artifact of assigning individuals inaccurate initial Elo scores that need to be corrected based on subsequently observed wins and losses. In Model 3, such compensations are not necessary because initial Elo scores are fitted to the observed data. To rule out the possibility that the lack of changes in individual Elo scores over time for females in Model 3 (*k* = 0) were driven by the low frequency of interactions among females, we generated 100 random subsamples of the male interaction data that matched the interaction frequencies observed for females, and repeatedly fitted Model 3 to each subsample. The estimated *k* values in these models ranged from 115.3–186.6 (mean: 143.5), indicating that results for model 3 were not an artifact of rare interaction frequencies. Overall, our findings indicate that there were no successful rank challenges and that therefore relative rank relationships among females did not change over their lifetimes. Instead, female chimpanzees of the Kasekela community queued for social status after hierarchy entry.

### Linearity of the female hierarchy

There is no formal method within the Elo rating framework for assessing whether there is a linear dominance hierarchy among the interacting individuals. However, because we found that dyadic dominance relationships among females did not change over time we were able to apply a matrix-based approach to assess hierarchy linearity, using one interaction matrix that spanned the entire study period (1969–2013). As some dyads in this matrix were unable to interact because the two individuals were not present in the group as adults at the same time, we applied the randomization test suggested by De Vries^46^ for interaction matrices with observational and structural zeros. We used this procedure to test for both linearity and non-linearity. Based on 100,000 randomizations, the test for linearity yielded a p-value of 0.079 and the test for non-linearity yielded a p-value of 0.921. These results are consistent with our working hypothesis that throughout most or all of our study period female chimpanzees formed a linear dominance hierarchy.

### Rank trajectories and determinants of entry ranks

**Figure 2:**
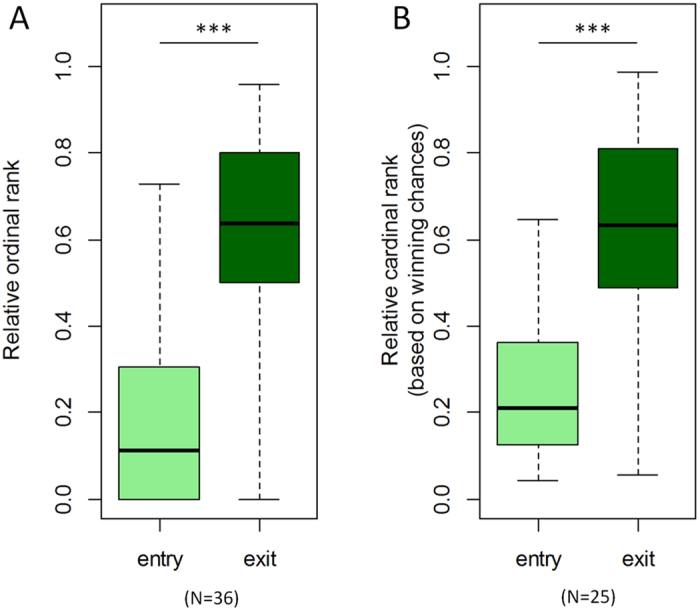
Difference in relative ranks at hierarchy entry due to immigration or maturation (N = 36) and exit due to emigration or death (N = 25). Boxplots indicate the minimum, 25% percentile, median, 75% percentile and maximum. Significant differences (p < 0.001) based on Mann-Whitney U tests are marked by stars (see text for details).

In the female rank trajectories as estimated by Model 3, no long-term changes in relative dyadic rank relationships occurred (Fig. 1f). Agonistic interactions in which the higher-ranked individual submitted or lost to the lower-ranked individual occurred in about 10% of interactions, but these interactions did not lead to long-term reversals of the established relative rank relationship between any two individuals. By extension, this means that long-term dominance rank trajectories among females after hierarchy entry were entirely controlled by changes in group composition, which is consistent with the hypothesis that female chimpanzees queue for social status. Although females tended to enter in the bottom half of the hierarchy and achieved a relatively high rank by the time they died (Fig. 2; *N*_*entry*_ = 36, *N*_*exit*_ = 25, Mann-Whitney U tests for relative ordinal rank: W = 102.5, p < 0.001, for relative cardinal rank: W = 97, p < 0.001), there was considerable variation among females in both entry and exit ranks. Queue jumping occurred, and jumps of at least one female at hierarchy entry were observed in 69% of all entries.

**Figure 3:**
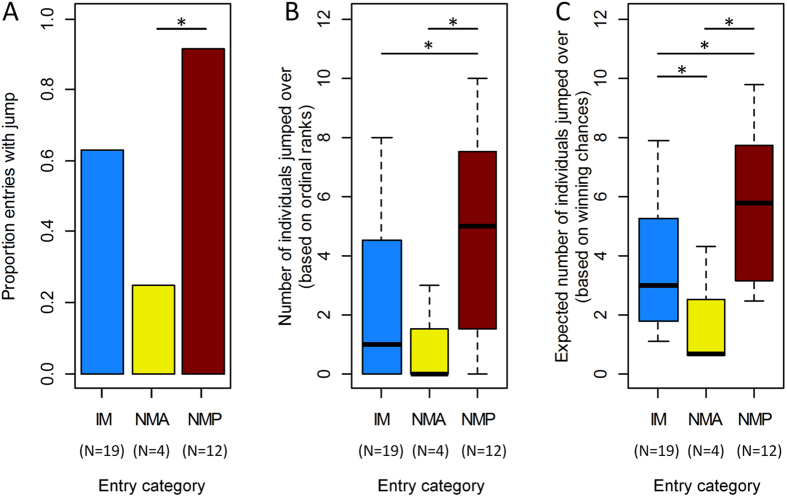
Illustration of queue jumping at entry into the hierarchy for different categories of individuals. IM: immigrants (N = 19); NMA: natal females with mother absent at time of entry (N = 4); NMP: natal females with mother present at time of entry (N = 12). Boxplots indicate the minimum, 25% percentile, median, 75% percentile and maximum. Significant pairwise differences (p < 0.05) based on Fisher’s exact tests and Mann-Whitney U tests are marked by a star (see Tables 2 and 3 for details).

Entry ranks differed significantly between females based on immigration status and presence of mothers at time of entry (Fig. 3, Tables 2 and 3). Both the frequency and distance of queue jumps was highest for natal females who matured into the hierarchy while their mother was still alive, and lowest for natal females whose mother was not alive at hierarchy entry. This pattern was consistent across multiple measures used to evaluate queue jumping (Fig. 3). Significant differences among all pairs of categories were only obtained for the measure that assessed “jump distance” based on the expected number of dominated individuals. Since this measure quantified variation in queue jumping in greatest detail, it is likely to have captured variation in queue jumping most accurately.

## Discussion

Our results document remarkable sex differences in the long-term dynamics of rank relationships among chimpanzees in the Kasekela community, indicating that females queue for social status while males actively compete for it. Specifically, we found that a) male rank trajectories are very dynamic, with males gaining and then losing rank by changes in relative rank relationships; b) females form stable long-term dominance relationships with each other, where successful rank challenges are rare and do not lead to long-term rank reversals. These findings contradict earlier impressions from Gombe that dominance relationships among female chimpanzees are either not clearly developed or are inconsistent^28^,^33^. They support the hypothesis that female chimpanzees queue for social status, as proposed by Nishida^36^ and also indicated by the tenure-based rank order found at Kanyawara^38^. The results of our test for hierarchy linearity are consistent with the linearity found in western chimpanzees^35^ and the sparse but transitive hierarchies published for Mahale^36^ and Budongo^47^.

The sex differences in the dynamics of rank relationships that we documented support expectations of rank trajectories based on sex-specific reproductive strategies common among many social mammals living in multi-male, multi-female groups. Reproductive skew among males is often linked to aggressive competition over access to females^6^. This results in dynamic individualistic hierarchies where relative rank relationships change over time due to differential resource holding potential^48^,^49^, sometimes aided by strategic alliances^18^,^50^. Male reproductive success in such dynamic hierarchies is partly determined by the extent to which males can balance the costs and benefits of acquiring and maintaining high rank^42^,^51^,^52^.

In comparison to males, female reproductive success is likely to be more strongly influenced by reproductive lifespan^53^,^54^. Thus, while females do compete over resources or mates, they are expected to generally gain fewer benefits from competing aggressively for dominance rank, either because alliance formation makes rank change difficult (as in many female cercopithecines^12^), or because of other mechanisms like size and age differences and winner-loser effects^55^ that maintain rank relationships once established. Further, any potential fitness gains associated with rank competition will be offset by the potential costs of injury for health, longevity, and offspring survival, all crucial aspects of female reproductive success in primates. Therefore, the tradeoffs in female dominance-related behavior should be skewed toward a tendency to maintain stable social relationships that facilitate social bonding and predictable interactions, patterns of behavior that have deeply rooted physiological underpinnings^56^.

The absence of long-term rank reversals among females implies that they are unable to compensate for (1) low entry ranks and (2) being “jumped over” by other females. This constrained ability to change ranks after hierarchy entry increases the importance of entering the hierarchy as high as possible; higher entry ranks can decrease the time needed to reach the top of the hierarchy and should therefore increase lifetime reproductive success, given the positive relationship between dominance rank and reproductive success in this community^30^. The conflict of interest between resident females and those entering the hierarchy, as reflected in often severe aggression against new immigrants^38^,^57^, could have promoted behavioral strategies and counterstrategies leading to the variation in entry ranks that we observed.

Among the potential factors that could determine rank at hierarchy entry, we found that the presence of mothers among natal females was associated with significantly higher entry ranks compared to immigrant females or natal females whose mothers had died by the time they entered the hierarchy. Given recent findings that mother-daughter pairs have, on average, the strongest social preferences among adult females in the Kasekela community^43^, kin support is a likely mechanism leading to the observed effect. Mothers could provide their daughters with a head start in the queue through coalition formation in agonistic interactions against lower-ranking females or because lower-ranking females are unlikely to challenge females due to the threat of alliance formation with their mothers. Although coalitions among females are rare at Gombe and elsewhere, they are more likely among resident females, including mothers and daughters, in aggression against immigrants^38^,^57^,^58^ and among closely bonded females in captivity^59^.

An alternative mechanism by which daughters of resident females might obtain higher entry ranks than immigrants is a potentially greater fighting ability caused by better access to high quality food resources. Daughters of higher ranking resident females are likely to forage in higher quality habitats if they share their mother’s core area^60^, and combined with their greater familiarity with local food distribution^61^ should have a generally higher foraging efficiency. The implied adaptive benefit of remaining in the natal community when mothers are still alive at time of hierarchy entry may be part of the explanation for why a higher proportion of females at Gombe fail to disperse^30^. However, such benefits should also exist at other study sites yet do not seem to prevent female dispersal at these sites as frequently as at Gombe. The small size of Gombe National Park, surrounded by unsuitable habitat, also limits options for dispersal and likely makes female philopatry a more favorable option for females at Gombe than elsewhere.

Although immigrant status and mother’s presence were significant predictors of average rank at hierarchy entry, entry ranks among both immigrant and natal females were highly variable. Potential explanatory factors for this variation, which have previously been associated with female chimpanzee dominance rank, include differences in age^30^, body mass^62^, body size^31^,^57^, and personality traits such as assertiveness^28^,^63^.

The apparent absence of long-term rank reversals among females raises the question of which mechanisms ensure this remarkable stability of rank relationships. Previous studies on other species revealed three potential mechanisms: 1) a lifelong increase in body size that could ensure that pairwise differences in fighting ability do not reverse over time; a mechanism consistent with patterns observed among African elephants^9^; 2) coalitionary support in agonistic interactions, as observed in male spotted hyenas (*Crocuta crocuta*)^14^; and 3) a feedback loop between fighting ability and dominance rank, where dominance rank is a function of body size, and where high-ranking individuals actively prevent lower-ranking individuals from body size increases^64^. Among female chimpanzees at Gombe, a similar feedback mechanism may contribute to the long-term stability of rank relationships; high-ranking females are known to gain access to better quality feeding areas^37^ and this could contribute to greater body mass as a result of improved access to food and feeding efficiency, particularly at times of food scarcity^62^. Further research is needed to assess both rank-related access to food and long-term stability of rank relationships at other study sites to determine sources of variation in the consistency of female chimpanzee social hierarchies and their functional significance.

## Additional Information

**How to cite this article**: Foerster, S. *et al*. Chimpanzee females queue but males compete for social status. *Sci. Rep.* **6**, 35404; doi: 10.1038/srep35404 (2016).

## Figures and Tables

**Table 1:** Overview and results of model fitting.

Model summary		Results for males (N = 22)			Results for females (N = 44)		
Model	Initial Elo scores	Estimated value of *k*	Δ AIC	Correctly predicted interactions	Estimated value of *k*	Δ AIC	Correctly predicted interactions
1	fixed at 0 for all individuals	137.0	0	95.1%	66.8	98.0	82.7%
2	forced to be at hierarchy bottom	130.3	29.6	95.4%	49.7	37.2	86.8%
3	fitted for all individuals	133.1	5.9	95.7%	<*0.001*	0	89.8%

**Table 2:** Results of Fisher’s exact tests of the frequency queue jumps for different categories of individuals.

Entry categories	p-value
IM vs. NMA	0.281
NMA vs. NMP	**0.027**
NMP vs. NMA	0.108

IM: immigrants; NMA: natal females with mother absent at time of entry; NMP: natal females with mother present at time of entry.

**Table 3:** Results of Mann-Whitney U tests of the distance of queue jumps for different categories of individuals.

	Number of individuals jumped over		Expected number of individuals jumped over	
entry categories	test statistic	p-value	test statistic	p-value
IM vs. NMA	53.5	0.202	64	**0.035**
NMA vs. NMP	6.5	**0.037**	5	**0.020**
NMP vs. IM	60.5	**0.029**	57	**0.020**

IM: immigrants; NMA: natal females with mother absent at time of entry; NMP: natal females with mother present at time of entry.
